# Supplementation with polyunsaturated fatty acids (PUFAs) in the management of attention deficit hyperactivity disorder (ADHD)

**DOI:** 10.1177/0260106018772170

**Published:** 2018-06-19

**Authors:** Tobias Banaschewski, Brendan Belsham, Michael H Bloch, Maite Ferrin, Mats Johnson, James Kustow, Sarah Robinson, Alessandro Zuddas

**Affiliations:** 1 Department of Child and Adolescent Psychiatry and Psychotherapy, Central Institute of Mental Health, Medical Faculty Mannheim, University of Heidelberg, Germany; 2 Private practice, Johannesburg, South Africa; 3 Child Study Center and Department of Psychiatry, Yale University, New Haven, USA; 4 Re:Cognition Health, London, UK; 5 University of Southampton, UK; 6 Gillberg Neuropsychiatry Centre, Sahlgrenska Academy, Göteborg University, Sweden; 7 Consultant Adult Psychiatrist and Adult ADHD specialist, London, UK; 8 UKAAN Executive board member and Chair of UKAAN’s Training Committee, London, UK; 9 PCM Scientific, London, UK; 10 Child and Adolescent Neuropsychiatry Unit, Department of Biomedical Science, Section of Neuroscience and Clinical Pharmacology, University of Cagliari, Italy

**Keywords:** Nutrition, ADHD, omega, health, supplements, attention deficit hyperactivity disorder, polyunsaturated fatty acids, PUFA

## Abstract

While pharmacotherapy and psychosocial interventions are recommended as the primary frontline treatment for attention deficit hyperactivity disorder (ADHD), alternative approaches to managing ADHD are becoming increasingly popular among patients and their families. Supplementation with polyunsaturated fatty acids (PUFAs) is an example of this. PUFA supplementation is not recommended by guidelines for managing ADHD; however, patients may still decide to use it. To provide direction to healthcare professionals (HCPs) managing ADHD, eight international experts in the field of adult and child ADHD came together for the Continuum Education Board: Omega Supplements in ADHD meeting. This commentary summarises the panel's consensus that current evidence suggests PUFA supplementation has a small beneficial effect on behaviour in children with ADHD, and that further high-quality research is needed to clearly evaluate and define its role in the management of ADHD of children, adolescents and adults. The panel concluded that in cases where patients use PUFA supplementation, HCPs should be comfortable explaining the potential gains that they may have and their possible side effects. The panel also concluded HCPs should not reinforce the idea that PUFA supplementation should replace treatment approaches with a more robust evidence base for managing ADHD.

## Introduction

Primary frontline treatments recommended for attention deficit hyperactivity disorder (ADHD) may make use of both pharmacotherapy and psychosocial interventions. However, alternative approaches to managing ADHD are becoming increasingly popular among patients and their families (Bos et al., 2015). Supplementation with polyunsaturated fatty acids (PUFAs) is one example of this; however, its use is not recommended by mainstream clinical guidelines, such as NICE guidelines (NICE guidelines, 2016), due to the relatively low effect size of meta-analyses’ results. Important questions such as how PUFA supplements should be used, if patients decide to use them, remain unanswered, underscoring the need for well-designed clinical trials to support healthcare professionals (HCPs) to make confident recommendations to their patients (Gow et al., 2015).

In an attempt to provide direction to HCPs managing ADHD, on 29 August 2017, eight international experts in the field of both adult and child ADHD came together for the Continuum Education Board: Omega supplements in ADHD meeting. The meeting served as a forum in which a panel of experts could assess the current landscape of PUFA supplementation in light of accumulating data, and provide practical recommendations to help HCPs make evidence-based decisions for patients and their families seeking alternative treatments. Details from the panel’s consensus are summarised here, along with recommendations for future research.

## Mechanism of action and rationale for effect in ADHD

Omega-3 PUFAs, such as eicosapentaenoic acid (EPA) and docosahexaenoic acid (DHA), are essential nutrients, and DHA is highly abundant in the mammalian brain (Bos et al., 2016). By altering cellular phospholipid membranes in the central nervous system, omega-3 PUFAs play an important role in different neural processes, some of which may be compromised in individuals with ADHD. Animal studies suggest effects on neurotransmission – via increased levels of serotonin and dopamine – and cell survival (Bazinet and Laye, 2014; Chalon, 2006). Omega-3 PUFAs might help minimise oxidative stress, which can be increased in individuals with ADHD (Bos et al., 2016).

Inflammation, which could be associated with neuropsychiatric disorders, might be reduced with EPA and DHA (Donev and Thome, 2010; Simopoulos, 2008). In contrast to omega-3 PUFAs, omega-6 PUFAs produce compounds that have pro-inflammatory properties (Königs and Kiliaan, 2016). Furthermore, a high omega-6/3 PUFA ratio is thought to have adverse health effects, including the development of some psychiatric disorders (Simopoulos, 2011). Indeed, Western diets show a trend towards higher omega-6/3 PUFA ratios, which has been posited to correlate with a higher rate of certain psychiatric conditions. The ratio between omega-3/6 PUFAs is important since omega-3 PUFAs competitively inhibit omega-6 PUFAs, causing a reduction in the synthesis of pro-inflammatory mediators (Ergas et al., 2002).

In line with these observations, recent findings have shown that some individuals with ADHD have low blood and plasma levels of omega-3 PUFAs (Bos et al., 2015; Chang et al., 2017; Parletta et al., 2016). Such a deficiency may have a significant and widespread impact on the function and development of the brain (Königs and Kiliaan, 2016). Therefore, increasing the intake of omega-3 PUFAs, through PUFA-rich diets or PUFA supplements, could be an appropriate strategy to address this potential deficiency and improve ADHD symptoms.

## Efficacy

PUFAs have been assessed across many psychiatric conditions. Meta-analyses of omega-3 PUFA trials in patients with depression have resulted in inconsistent conclusions. One analysis concluded that omega-3 PUFA supplementation does not have a significant benefit on depressive symptoms (Bloch and Hannestad, 2012). Meanwhile, several others have found significant beneficial effects of EPA alone on depression (Hallahan et al., 2016; Martins et al., 2012; Mocking et al., 2016). In schizophrenia and other psychotic disorders, current data are inconclusive for omega-3 PUFAs. (Akter et al. 2012; Freeman et al., 2006; Fusar-Poli and Berger, 2012). Promising results from one study suggest omega-3 PUFA supplementation reduces the risk of progression to psychotic disorders and psychiatric morbidity (Amminger et al., 2015). Data from autism (van Elst et al., 2014), anxiety disorders (Ravindran and da Silva, 2013) and obsessive–compulsive disorder studies (Fux et al., 2004) are too sparse from which to draw conclusions.

In ADHD, clinically meaningful effect sizes have been observed in some studies using PUFA supplements. Several prospective, interventional studies have suggested that omega-3/6 PUFA supplements – either alone or in combination – offer improvement in cognitive performance (Sinn et al., 2008) and enhanced tolerability of the stimulant methylphenidate when prescribed as an add-on treatment (Barragán et al., 2017; Sinn et al., 2008). One report describes improvement in measures that include sleep quality and emotional functioning (Chen et al., 2004). In recent years, attempts to clarify the effect of PUFA supplementation in ADHD have culminated in the publication of several meta-analyses, all relating to children/adolescents (Bloch and Qawasmi, 2011; Chang et al., 2017; Gillies et al., 2012; Sonuga-Barke et al., 2013).

Meta-analyses mainly report a small effect size in ADHD (Table 1), although they differ in how the clinical benefit is interpreted. In the Cochrane review by Gillies et al. (2012), PUFA supplementation showed a small, non-significant impact on ADHD symptoms (parent-ratings: overall symptoms (standardised mean difference (SMD): 0.17); inattention (SMD: 0.04); hyperactivity/impulsivity (SMD: 0.04) and teacher-ratings: overall symptoms (SMD: –0.05); inattention (SMD: –0.26); hyperactivity/impulsivity (SMD: –0.10)). The authors concluded that, overall, there was no significant benefit for PUFA supplementation in children and adolescents with ADHD compared with placebo. Meanwhile, Bloch and Qawasmi (2011) found a small but statistically significant improvement on ADHD symptoms with omega-3 PUFA supplementation (particularly with EPA) compared with placebo (overall ADHD symptoms (SMD: 0.31); inattentive symptoms (SMD: 0.29); hyperactivity (SMD: 0.23)), equating to small efficacy compared with existing pharmacotherapies, such as psychostimulant medication (Bloch and Qawasmi, 2011). Sonuga-Barke et al. (2013) found that PUFA supplementation produced small but statistically significant reductions in overall ADHD symptoms (SMD: 0.21). When ‘probably blinded’ assessments were used, effects remained statistically significant for overall symptoms (SMD: 0.16); however, when stratified into specific aspects of ADHD, the effect sizes for each component were: inattention, SMD: 0.11; hyperactivity/impulsivity, SMD: 0.13. In a recent systematic review and meta-analysis, Chang et al. (2017) suggested that monotherapy with omega-3 PUFA supplementation does benefit children and adolescents with ADHD compared with placebo, and is associated with an improvement in both their clinical symptoms (Hedges’ *g* effect size (*g*): 0.38, *p*<0.0001) and their cognitive performance (*g*: 1.09, *p*=0.001).^12^

**Table 1.** Summary of meta-analyses assessing efficacy of PUFA supplementation in children and adolescents with ADHD.

Study	*N*	Intervention	Studies included	Effect size (overall ADHD symptoms)
Chang et al.	534	Omega-3 PUFAs	Placebo-controlled	*g*: 0.38
Bloch and Qawasami	699	Omega-3, omega 3/6 PUFAs	Placebo-controlled	SMD: 0.31
Gillies et al.	1011	Omega-3/6 PUFAs	Placebo-controlled and cross-over	SMD: 0.17 (parent-rated) SMD: –0.05 (teacher-rated)
Sonuga-Barke et al.	890	Omega-3, omega-6, omega-3/6 PUFAs	Placebo-controlled and cross-over	SMD: 0.21 (m-proxy) SMD: 0.16 (p-blind)

ADHD: attention deficit hyperactivity disorder; *g*: Hedges’ *g* effect size; PUFA: polyunsaturated fatty acid; SMD: standardised mean difference.

Variations in the inclusion criteria for meta-analyses may account for these conflicting results in terms of significance or non-significance. It should be noted that Cochrane reviews are stricter in terms of what data they will typically include to calculate effect size, so fewer studies usually contribute to the meta-analysis. It should also be noted that studies included also vary in sample size, trial length, dosage, supplement composition and whether participants are concurrently taking stimulant medication. In summary, current evidence suggests that PUFA supplementation has a small beneficial effect on behaviour in children with ADHD.

## Further considerations

Although effect sizes on ADHD symptoms are small compared with established stimulant treatments, research suggests there are virtually no severe side effects of PUFA supplements. The most frequently reported side effects are dyspepsia and incidental nosebleeds (Königs and Kiliaan, 2016).

In addition, there have been reports of PUFA supplement products being highly oxidised, with oxidation levels exceeding recommended averages (Albert et al., 2015). This is often not considered in trials and may account for their inconsistent results.

Research has also suggested that certain subsets of patients (possibly those with developmental disorders and other comorbid conditions, and patients with PUFA-poor diets) may stand to benefit more than other groups of patients (Chang et al., 2017; Parletta et al., 2016). It has been postulated that this may reflect the heterogeneity of the underlying causes of ADHD, for example, a deficiency in PUFAs or certain food intolerances (Chang et al., 2017).

## Proposed future research

It is evident that further high-quality research is needed to clearly evaluate and define the role of PUFA supplementation in the management of ADHD for children, adolescents and adults (Gow et al., 2015). First, it is crucial that future trials should include larger samples. As the benefits of omega-3 PUFA supplements are likely to be small, larger samples will be needed to demonstrate a statistically significant effect. It is worth considering that even a small improvement from omega-3 PUFA supplements may be clinically useful given the absence of significant side effects and the growing evidence that they may be health-promoting in other areas of medical and psychiatric health (Amminger et al., 2015; Bos et al., 2016).

Blood PUFA levels should be routinely analysed and monitored in future trials as it would allow researchers to study compliance, likely responders, and correlations between PUFA blood levels and ADHD symptoms. Furthermore, circumstantial findings suggest that individuals with certain dietary deficiencies may stand to benefit more from PUFA supplementation, yet, to our knowledge, no prospective investigation has taken place to test this hypothesis, and it should be a focus of future research. This information may enable patient stratification in the future.

Additionally, future research should aim to clarify the difference in efficacy between omega-3 and omega-3/6 combination PUFA supplements. Future clinical trials should also include data on oxidation values as well as average measured contents of EPA and DHA in the supplements used in the studies.

Much interest has been generated in identifying patient populations more likely to respond to PUFA supplementation. One study has suggested that PUFA supplements might have a higher impact on a subgroup of patients with the inattentive presentation and other neurodevelopmental problems (Johnson et al., 2009). To this end, data collected from future trials should include information on concomitant use of other nutritional supplements that may have a masking effect on PUFAs, and also on comorbid conditions and the gender of the participants.

Other unanswered questions exist regarding the optimal age in which to use PUFA supplements. In animal studies, where there is an existing omega-3 PUFA deficiency, it has been shown that maternal supplementation during the pre-/post-natal period restores processes affected by the deficiency if given prior to the 21st day of life (Kodas et al., 2004), suggesting an optimal time frame may exist in humans too. Thus, future studies should also focus on the prophylactic impact of PUFA supplementation in the pre-natal and neonatal period. It is also crucial to encourage research on PUFA supplements in adults with ADHD as most research presently focuses on children and adolescents. Finally, more research should be carried out to assess the optimal duration of PUFA treatment.

## Recommendations for HCPs communicating with ADHD patients and their families

Interest in PUFA supplementation as a possible treatment for a wide range of diseases and conditions has increased remarkably among the public and the media. Their growing popularity is likely attributable to their tolerable safety profile and concerns about the over-medicalisation of children.

As a result, HCPs are increasingly asked by patients and their families about the role of PUFA supplements in neurodevelopmental disorders, such as ADHD. Commonly, PUFA supplements are requested by those who are not keen to use, would like to delay using or have not tolerated more traditional pharmacological treatments for ADHD. Many of these patients and families want to try PUFA supplementation as a first step, before starting a stimulant or other ADHD treatments. Some individuals request to use them in combination with stimulant treatment, either because they believe this will enhance the therapeutic effect or improve tolerability.

In these scenarios, HCPs should ensure patients and their families are aware that, in all likelihood, PUFA supplements typically do not have as much effect on ADHD symptoms as stimulants but that they are generally well tolerated. HCPs should not necessarily advise patients and families against using PUFA supplements, so long as their use does not deter them from using first-line treatments which have a stronger evidence base. It is important to mention that PUFA supplements have been associated with minor dyspepsia.

HCPs should also advise patients and their families to try to find the purest source of PUFA supplements available, ensuring that the preparation contains EPA, DHA and vitamin E (added to prevent oxidation of fatty acids) and that, ideally, they contain no flavours or colours. In situations where a patient wishes to use a PUFA supplement instead of stimulant medication, HCPs should advise them to take at least 750 mg of both EPA and DHA per day for at least 12 weeks before evaluating the response (recommendation of the expert panel). HCPs should discuss with patients and their families the available clinical evidence for PUFA supplements and other treatments to ensure they are making an informed choice.

## Conclusion

PUFA supplementation, in particular omega-3 PUFAs, may produce small but statistically significant reductions in ADHD symptoms while having a tolerable safety profile. Accumulating evidence suggests that they may offer benefits outside of ADHD symptom control, including improvements in sleep quality and cognitive function, but more research is needed to confirm these additional benefits. The variation in results from the meta-analyses conducted, however, highlights the need for caution when interpreting studies. Moreover, HCPs should consider each patient on an individual basis, taking into account individual preference, current ADHD severity and treatment history before discussing PUFA supplementation. In cases where PUFA supplements are used, HCPs should be comfortable explaining the potential gains that PUFA supplementation may have and its possible side effects. Furthermore, HCPs should not reinforce the idea that PUFA supplementation should replace treatment approaches with a more robust evidence base for managing ADHD.
